# Probiotic potential of enterococcus lactis in improving egg production and quality in quails during late egg-laying period

**DOI:** 10.1016/j.psj.2025.104765

**Published:** 2025-01-01

**Authors:** Lan Liu, Changcai Liu, Huixiang Wang, Hao Tang, Zhe Chen, Xufeng Dou, Jiaxin Chang, Zhengxing Li, Zhichao Wang, Yuxia Mei, Min Ren

**Affiliations:** a Key Laboratory of Conservation and Utilization of Biological Resources in the Tarim Basin, Alar, Xinjiang 843300, China; b College of Life Science and Technology, Tarim University, Alar, Xinjiang 843300, China; c College of Animal Science and Technology, Tarim University, Alar, Xinjiang 843300, China; d Xinjiang Aksu region Wensu County moss flower farming and animal husbandry technology Co., Ltd., Aksu, Xinjiang, China; e College of Life Science and Technology, Huazhong Agricultural University, Wuhan 430000, China

**Keywords:** Quail, Enterococcus lactis, Probiotic properties, Egg production, Egg quality

## Abstract

This study investigated the probiotic potential of lactic acid bacteria (LAB) strains isolated from the intestines of quails during the late egg-laying period. Eight LAB strains were examined for their tolerance to acid and bile salts, antibiotic susceptibility, self-aggregation, hydrophobicity, and antibacterial activity. Among these, *E. lactis* TRM58998 demonstrated the most favorable characteristics, including robust resistance to acid and bile, as well as significant antimicrobial properties. In a 30-day feeding trial, 96 quails (43 weeks old) were divided into two groups: a control group receiving a basal diet and a treatment group supplemented with 1.0 × 10^8^ CFU/g of *E. lactis* TRM58998. The quails in the probiotic group showed a significant increase in egg production, average egg weight (P < 0.05), and enhanced eggshell quality (P < 0.01). Additionally, the treatment improved antioxidant capacity and stimulated ovarian follicle development, as indicated by elevated follicle-stimulating hormone (FSH) levels (P < 0.05). These findings suggest that *E. lactis* TRM58998 can effectively enhance quail performance, egg quality, and antioxidant status, underscoring its potential for promoting sustainable practices in quail farming.

## Introduction

With increasing restrictions on antibiotics in poultry feed, identifying effective alternatives to promote animal health and productivity has become essential. Probiotics, defined as live microorganisms that provide health benefits to the host, have emerged as a promising solution in poultry farming. They improve immune function, regulate gut microbiota, and enhance nutrient absorption, contributing to better feed efficiency and reduced disease incidence (Fonseca et al., 2024). Probiotics are live microorganisms. It is now considered a suitable alternative to antibiotics to fight animal infections and improve animal production (El-Hack et al., 2022). More than ten genera such as *Lactobacillus, Bacillus, Enterococcus, Bacteroides, Streptococcus, Lactococcus* and *Bifidobacterium* are widely studied for their probiotic applications (Xia et al., 2024). Among the various probiotics, Lactic acid bacteria (LAB) are known for producing antimicrobial substances, including organic acids and bacteriocins, that inhibit pathogenic bacteria by competing for nutrients and attachment sites (Niibo et al., 2019). They also adhere to the gastrointestinal tract, forming a protective barrier that regulates immune responses, strengthens the intestinal mucosa, and prevents the invasion of harmful substances (Fentie et al., 2024). This action supports host immunity and improves intestinal microflora (Dai et al., 2020; Hui et al., 2024), Among LAB, *Enterococcus faecalis* is particularly effective at promoting intestinal health. Studies have shown that incorporating *E. faecalis* into the diet reduces intestinal damage, enhances gut microbiota regulation, and strengthens immune responses in broilers (Wu et al., 2019). Furthermore, *E. faecalis* supplementation has been found to alleviate pathogen-induced damage, emphasizing its role in protecting intestinal health and regulating immunity (Huang et al., 2022). *E. faecalis* is widely recognized for its positive impact on gut health, but it is *Enterococcus lactis* that truly shines with its unique metabolic benefits, including significant cholesterol reduction in animal models, positioning it as a leading probiotic candidate (Kriti et al., 2022).

Originally isolated from milk (Morandi et al., 2012), *E. lactis* has captured scientific attention for its ability to produce bacteriocins and its exceptional probiotic qualities. *E. lactis* PMD74 has been identified as a potential probiotic and has shown the ability to inhibit the growth of Gram-positive bacteria (Tezel, 2019); Recent studies demonstrate that *E. lactis* is a safe and effective alternative to *E. faecalis* in the food industry (Lu et al., 2023). This remarkable bacterium not only exhibits strong antibacterial properties and heat resistance, but it also shows promise in detoxifying mycotoxins in animal feed (Tadesse et al., 2024; Yao et al., 2024). Importantly, *E. lactis* has been proven to enhance lipid metabolism and lower serum cholesterol levels, highlighting its robust probiotic characteristics across diverse environments, including the human gut, dairy products, and marine ecosystems (Braïek et al., 2018; Guo et al., 2016). Moreover, its flexibility as a feed additive for livestock translates to improved productivity and health outcomes in piglets, broilers, and hens (Bampidis et al., 2023). With its vast potential benefits, *E. lactis* is an invaluable addition to both human diets and animal nutrition.

The versatility of *E. lactis* across various animal systems reveals its significant potential to tackle the unique challenges of poultry farming. Quail farming, an emerging and dynamic sector within this industry, faces critical challenges that demand immediate attention. Ensuring the health and productivity of egg-laying quails, particularly during the late laying phase, is essential for success. Short peak laying periods, metabolic imbalances, calcium depletion, reduced egg production, and poor eggshell quality can result in substantial economic losses (Dai et al., 2020; Fu et al., 2024). Probiotics, especially host-specific strains, have shown remarkable efficacy in addressing these issues by improving nutrient digestion, enhancing immunity, and maintaining intestinal health (Jose et al., 2015). Research clearly demonstrates that the ideal probiotics must originate from the gastrointestinal tract of animals for maximum effectiveness (Carnevali et al., 2014; Lazado et al., 2015). However, the potential of *E. lactis* in quail farming is still largely untapped, particularly during the critical late laying period. This underscores an urgent need for research into host-adapted probiotic strains that specifically meet the physiological needs of quails, to maximize their health and productivity during these demanding production phases. Investing in this research could revolutionize quail farming and contribute to greater economic stability within the industry.

This study focused on isolating LAB strains from the cecum and feces of quails to evaluate their probiotic properties. We tested the strains for acid and bile salt tolerance, antibiotic sensitivity, and bacteriostatic capacity to identify those that enhance quail performance. Feeding experiments assessed their impact on egg production and intestinal health in the later stages of laying. The findings aim to support the development of customized probiotic solutions for sustainable quail farming and increased productivity.

## Materials and method

### Sources of bacterial samples

Intestinal contents and feces from quail were utilized for strain isolation. Healthy, 12-week quails were sourced from Wensu County Moss Flower Farming and Animal Husbandry Technology Co., Ltd. in the Aksu region of Xinjiang. An expert and a veterinarian evaluated their breed and health during the peak laying period, and the quail had not been given any antibiotics.

### Bacterial isolation and identification

Quails were bled and euthanized in the Engineering Laboratory of Tarim Animal Diseases Diagnosis and Control, Xinjiang Production & Construction Corps, and their body surface was disinfected with 75 % ethanol. The cecum tissue was quickly dissected, and the ends were tied tightly using sterile cotton thread to ensure that the contents of the cecum were not exposed to the air. The cecum was cut just before the ligation, quickly placed into an ice box, and transported back to the laboratory. In the laboratory's sterile workstation, the ligated part of the cecum was cut off, and the contents of the cecum were squeezed into a sterile 5mL centrifuge tube using sterile forceps. (Shen et al., 2024; Zhu et al., 2023); To ensure the integrity of the fecal samples and prevent contamination or other bacterial growth, only fresh feces were used and collected in sterile 50 mL centrifuge tubes, and immediately transferred to an ice box to ensure they arrived at the laboratory in optimal condition for further processing.

To isolate the bacterial strains, 2 g of each cecum sample and fecal were weighed in a centrifuge tube and incubated with 18 mL of sterile water for 20 min at 37 °C in a shaker at 200 r/min. The supernatant was then diluted in a gradient, and 10^−5^, 10^−6^, and 10^−7^ were applied to MRS and TSB media, respectively (Shen et al., 2024). The media were incubated in a 37 °C incubator for 24 h. The colonies on the media were picked and isolated by multiple streaking until purified. The colonies on the media were picked and isolated by multiple streaking until purified. The 16S rDNA sequences were sequenced by Sanger sequencing, and the sequencing results were compared to confirm the identity of the bacterial strains before they were conserved.

### In vitro characterization of probiotic properties of the isolates

*Acid and Bile Salt Resistance Assays* Referring to the method of Manzoor et al (Manzoor et al., 2019), with slight modifications. Bacterial isolates were suspended in MRS broth adjusted to pH 2.0 and 4.0 and in media containing 0.03 % and 0.3 % bile salts (Shanghai McLean Biochemical Technology Co., Ltd.). Cultures were incubated at 37 °C for 3 h, and viable counts were determined by plating on MRS agar.

*Hydrophobicity Assay* Refer to the method established by Crow et al (Crow et al., 1995). Combine suspensions of 10⁸ CFU/mL with chloroform in a 3:1 ratio and incubate the mixture at 37 °C for 30 min. After incubation, measure the absorbance of the aqueous phase at OD600. Record the absorbance at 0 min as G1 and at 30 minutes as G2 to calculate the hydrophobicity rate using the following formula (1):

(1) $$\text{Hydrophobicity}\;\text{Assay}\;\%=\frac{G1-G2}{G1}\times \;100$$

*Auto Aggregating Capacity* Refer to the method by Magdalena et al (Magdalena et al., 2014). A suspension of 10^8^ CFU/mL was centrifuged, washed with PBS, and the absorbance in OD600 was adjusted to 0.5. It was then incubated at 37 °C for 6 h. The self-aggregation rate was measured by recording the absorbance at 0 h (OD0) and at 6 h (OD60), and the auto-aggregation rate was calculated using the formula (2):

(2) $$\text{Auto}\;-\text{aggregation}\;\text{rate}\;\%=\frac{\mathrm{OD}0-\mathrm{OD}60}{\mathrm{OD}0}\times \;100$$

*Antimicrobial Activity* Antimicrobial activity was tested against *Escherichia coli* ACTCC 25922, *Staphylococcus aureus* ATCC 6538, four strains of *Senteritidis* (D58-1, D58-2, D62-3, and F21) of sheep origin, *Pseudomonas aeruginosa* ACTCC 27853, *Acinetobacter baumannii* ACTCC 19606, *Shigella flexneri* ATCC 12022, *Candida albicans* ATCC 25923, *Salmonella enteritidis* ACTCC 25923, and *Klebsiella pneumoniae* ATCC 10031 using the Oxford Cup method. Inhibition zones were measured with a vernier calliper after 24 h of incubation at 37 °C.

*Antibiotic Susceptibility Test* Susceptibility disk diffusion method was used to determine the antibiotic sensitivity of the strains. Observe and measure the diameter of the inhibition zone. The diameter of the inhibition zone reflects the resistance of the strain to antibiotics. The evaluation criteria refer to the American Society for Clinical and Laboratory Standard (Reuben et al., 2019). The antibiotics included were; Ciprofloxacin (5 ug/disk), Ceftriaxone (40 ug/disk) , Ampicillin (10 ug/disk), Erythromycin (15 ug/disk), Chloramphenicol (30 ug/disk), Penicillin(10 ug/disk), Tetracycline (30 ug/disk), Gentamicin (10 ug/disk), Lincomycin (2 ug/disk), Compound Sulfamethoxazole (25 ug/disk).

*Hemolysis test* The hemolytic activity was assessed according to Cui et al. (Cui et al., 2018), Isolate cultures were streaked onto nutrient agar medium supplemented with 5 % sheep blood, then incubated at 37 °C for 48 h. The plates were later examined for signs of red blood cell lysis surrounding the bacterial colonies.

### Animals and experimental design

Ninety-six quails, aged 43 weeks, were randomly selected from a commercial flock for this study. They were housed in cages with continuous access to feed and water, maintained at a temperature of 29 ± 3 °C, and provided with 16 h of light per day, all in accordance with the regulations set by the Animal Ethics Committee (2024072) at Tarim University.

The study evaluated the effects of probiotics on egg production and intestinal health over a 30-day feeding experiment. Prepared probiotic powders according to the method of Phoomjai et al. (Phoomjai et al., 2022) and quantified the number of live bacteria using plate counting methods. The quails were randomly and equally divided into two groups of three parallels each with 16 quails each: one received a basal diet (25 g) supplemented with 1.0 × 10^8^ CFU/g probiotics, while the other received only the basal diet. The feeding experiment followed the protocols described by Nour et al (Nour et al., 2021), with separate cages assigned to each treatment group to prevent diet mixing.

*Feed Mixing Procedures and Egg Collection* Quails were fed twice daily at 9:30 AM and 6:30 PM, with weekly recordings of egg weight and feed intake.

The day prior to the experiment, 12 quails (3 from each replicate) were randomly selected and euthanized. Blood samples were collected and centrifuged at 4 °C at 3000 rpm for 15 min to separate the serum (Li et al., 2024), which was then frozen at -20 °C for subsequent biochemical and antioxidant analyses. The ovaries were also collected and stored at 4 °C (Chen et al., 2016).

*Laying performance* Quail eggs were collected each day at the same time, and data on the number of eggs, their weights, and daily feeding amounts were recorded. This information was then utilized to calculate the weekly egg production rate, average egg weight, and average daily feed intake.

(3) $$\text{Egg}\;\text{production}\;\text{rate}\;\left(\%\right)=\frac{\mathrm{Total}\;\mathrm{number}\;\mathrm{of}\;\mathrm{eggs}}{\mathrm{Number}\;\mathrm{of}\;\mathrm{quails}\;\times \;\mathrm{Number}\;\mathrm{of}\;\mathrm{days}}\times \;100$$

(4) $$\text{Average}\;\text{egg}\;\text{weight}\;\left(g\right)=\frac{\mathrm{Total}\;\mathrm{egg}\;\mathrm{weight}}{\mathrm{Total}\;\mathrm{number}\;\mathrm{of}\;\mathrm{eggs}}$$

(5) $$\text{Average}\;\text{daily}\;\text{feed}\;\text{intake}=\frac{\mathrm{Total}\;\mathrm{feed}\;\mathrm{intake}}{\mathrm{Number}\;\mathrm{of}\;\mathrm{quails}\;\times \;\mathrm{Number}\;\mathrm{of}\;\mathrm{days}}$$

(6) $$\text{Feed}-\text{to}-\text{egg}\;\text{ratio}\;\left(\%\right)=\frac{\mathrm{Total}\;\mathrm{feed}\;\mathrm{consumption}}{\mathrm{Egg}\;\mathrm{production}}$$

*Egg quality* Egg quality was determined using the method of Nour et al. (Nour et al., 2021); Egg quality analysis was performed weekly, and vernier callipers measured the length and width of quail eggs. The egg shape index is calculated as egg length/egg width. Eggshell strength was measured by KQ-1A eggshell strength tester (Beijing Tianxiang Feiyu Technology Co., Ltd.). Yolk height was measured using an EQ-1A mini-type egg white height tester (Beijing Tianxiang Feiyu Technology Co., Ltd., Beijing, China). The yolk index was calculated as the yolk height divided by yolk diameter (yolk height (mm) / yolk diameter (mm)) × 100 %; the yolk proportion is calculated as (yolk weight (g) / egg weight (g)) × 100 %. Haugh Unit (HU) scores are calculated according to the following formula:

(7) $$\begin{array}{c} \text{Hu}\left(\%\right)=100\times \text{log}\left(H-W^{0.37}+7.6\right) \end{array}$$

Where H and W refer to albumen height and egg weight, respectively.

*Serum biochemical assay, Antioxidant Parameters and serum hormone* Serum biochemical indicators, including albumin (ALB), total protein (TP), globulin, alanine aminotransferase (ALT), aspartate aminotransferase (AST), urea nitrogen (BUN), amylase (AMY), alkaline phosphatase (ALP), creatinine (Crea), glucose (GLU), triglycerides (TG), total cholesterol (TC), calcium (Ca), and inorganic phosphorus (PHOS), The test was conducted using a fully automated multifunctional biochemistry analyzer (SMT-120VP from Chengdu Smartech Co., Ltd., China) and a healthy 16-item quantitative test reagent panel (Chengdu Smartech Co., Ltd., China) 0.1 mL of separated plasma was taken, added to the reagent disk, placed into the detector, and tested according to the instructions provided. Finally, the data were recorded.

Malondialdehyde (MDA), glutathione peroxidase (GSH-Px), superoxide dismutase (SOD), and catalase (CAT) levels in serum were measured according to the protocols provided by Suzhou Grace Biotechnology Co., Ltd.

Follicle-stimulating hormone (FSH), luteinizing hormone (LH), and estradiol (E2) levels were determined with a kit from Jiangsu Jingmei Technology Co., Ltd.

*Follicle number* Follicle diameters were measured using vernier calipers and classified accordingly (Pengfei et al., 2017). Preovulatory follicles were defined as those exceeding 12 mm, small yellow follicles ranged from 6 to 8 mm, and large white follicles had diameters between 3 and 5 mm.

*The Analysis of Cecal Microbiota* The cecal contents were processed for DNA extraction and high-throughput 16S rDNA sequencing. Sequencing was performed by Shanghai Paisenno Biotechnology Co., Ltd. using an Illumina platform. Data analysis took place on the Paisenno Cloud platform to evaluate microbial diversity and composition. (https://www.genescloud.cn/login).

### Data analysis

Data analysis in this study were subjected to independent samples t-test using SPSS 22.0, with results presented as mean ± SEM (standard error of mean), with significance set at P < 0.05. Graphs were generated using OriginPro 2022.

## Results

### Composition of intestinal bacteria and candidate probiotics

**Fig. 1:**
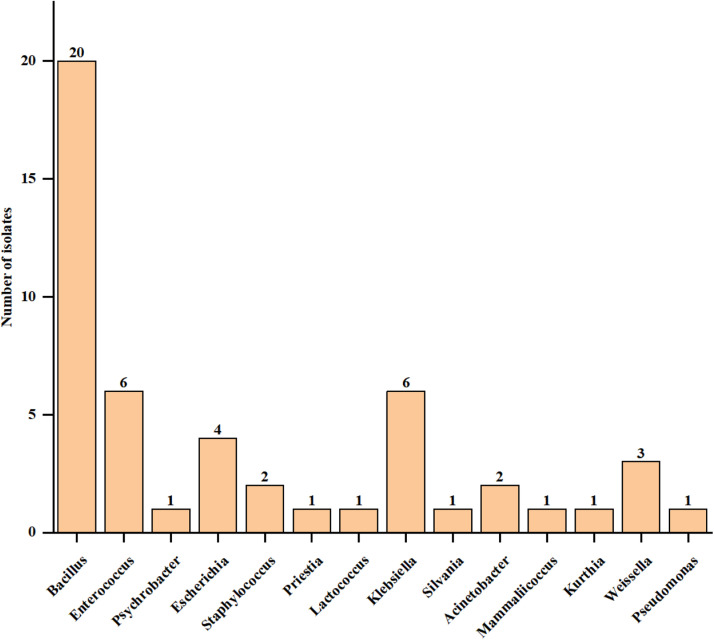
Composition of enteric bacteria at the genus level.

A total of 52 bacterial strains were isolated from the intestines and feces of quails, primarily belonging to the genera *Bacillus, Enterococcus*, and *Klebsiella*, which collectively accounted for 64 % of the total. Among these, 8 strains were identified as lactic acid bacteria (LAB) from four genera: *Enterococcus, Lactococcus, Weissella*, and *Pediococcus*, representing 16 % of the total isolates. Further details can be found in (Table 1 and Fig. 1).

**Table 1.** Information about lactic acid bacteria isolated from the intestinal contents and feces of quail.

Strain No	Sequence length	Species	Similarity	GeneBank
TRM58999	1446 bp	*Enterococcus faecium*	99.65 %	PP422246
TRM58998	1445 bp	*Enterococcus lactis*	99.72 %	PP422247
TRM58997	1447 bp	*Loigolactobacillus coryniformis subsp. torquens*	99.79 %	PP422248
TRM58996	1444 bp	*Enterococcus faecalis*	99.38 %	PP422249
TRM58995	1462 bp	*Pediococcus pentosaceus*	99.93 %	PP422250
TRM58994	1459 bp	*Weissella hellenica*	99.93 %	PP422251
TRM58993	1460 bp	*Weissella paramesenteroides*	99.45 %	PP422252
TRM58992	1464 bp	*Weissella sagaensis*	100 %	PP422253

### In vitro characterization of probiotic properties of the isolates

*Acid and bile salt tolerance* All strains could grow at pH levels of 2.0 to 4.0 and bile salt concentrations of 0.03 % to 0.3 %. Notably, TRM58998 and TRM58995 showed strong resistance to prolonged acid and bile exposure (Table 2).

**Table 2.** Lactic acid bacteria strains acid and bile salt tolerance.

Strain No.	Survival percentage at different pH values (%)		Survival percentage at different times and bile salt concentrations (%)	
	pH2.0	pH4.0	0.03 %bile salt	0.3 %bile salt
TRM58999	81.890 ± 0.241	76.423 ± 0.643	81.975 ± 1.169	62.353 ± 0.551
TRM58998	85.426 ± 0.295	87.784 ± 0.260	87.533 ± 0.784	82.912 ± 0.323
TRM58997	74.811 ± 2.072	83.824 ± 0.514	71.666 ± 2.335	67.021 ± 4.773
TRM58996	81.113 ± 0.355	85.991 ± 0.152	70.565 ± 1.609	73.691 ± 0.400
TRM58995	83.114 ± 0.954	81.822 ± 0.590	73.105 ± 1.623	64.733 ± 1.225
TRM58994	81.481 ± 0.608	82.308 ± 0.178	69.084 ± 0.887	65.465 ± 3.235
TRM58993	82.000 ± 0.938	82.525 ± 0.313	69.410 ± 1.209	64.300 ± 1.440
TRM58992	78.430 ± 0.637	80.710 ± 0.518	66.070 ± 1.556	62.990 ± 2.013

Note: The data represents the survival rate of bacterial strains after treatment under different conditions for 3 h.

*Auto aggregation ability and hydrophobicity of the LAB strains* The auto-aggregation capacities of eight strains ranged from 48.361 % to 60.770 %, with TRM58992 achieving the highest value. Likewise, the cell surface hydrophobicity measurements varied between 14.023 % and 52.875 %, with TRM58992 once again demonstrating the highest level at 52.875 % (Table 3).

**Table 3:** Auto aggregation ability and hydrophobicity of lactic acid bacteria strains.

Strain No.	Auto aggregation ability and Hydrophobicity	
	Auto aggregating ability (%)	Hydrophobicity (%)
TRM58999	58.332 ± 0.555	42.210 ± 3.739
TRM58998	56.161 ± 0.878	47.907 ± 1.864
TRM58997	54.674 ± 0.641	35.283 ± 0.182
TRM58996	49.775 ± 1.226	38.002 ± 2.461
TRM58995	55.634 ± 1.172	48.205 ± 0.885
TRM58994	48.361 ± 1.004	49.107 ± 3.404
TRM58993	54.587 ± 0.291	14.023 ± 5.468
TRM58992	60.770 ± 1.407	52.875 ± 3.269

Note: The Auto aggregating ability (%) represents the survival rate of bacterial strains after treatment under conditions for 6 h; The Hydrophobicity (%) represents the survival rate of bacterial strains after treatment under conditions for 30 min.

*Antimicrobial Activity of Isolates Against Pathogenic Test Organisms* We assessed the inhibitory effects of eight strains on twelve microbial pathogens. All strains demonstrated inhibitory activity against *Escherichia coli* and two strains of *Salmonella*. Notably, TRM58998, TRM58995, and TRM8994 exhibited significant inhibition, with TRM58998 and TRM58995 showing inhibition zones exceeding 15 mm, as detailed in (Table 4).

**Table 4:** Antibacterial activity of lactic acid bacteria strains against twelve pathogenic strains.

Strain No.	Inhibition zone diameter (mm)											
	EC	Sal	Sal D58-1	Sal D58-2	Sal D62-3	Sal F21	Pa	Kp	Ab	Sf	Sa	Ca
TRM58999	13.540	13.090	-	12.060	12.700	13.180	-	-	12.960	-	12.100	13.670
TRM58998	22.220	17.620	6.390	19.930	19.400	20.500	19.070	15.660	17.080	23.490	21.300	22.300
TRM58997	18.250	12.960	11.150	13.690	14.060	16.080	13.720	11.950	24.100	-	14.740	12.770
TRM58996	15.770	15.630	-	21.530	23.480	20.160	-	12.030	15.720	15.200	15.280	-
TRM58995	21.490	24.660	27.950	27.510	29.250	23.020	28.870	21.430	26.970	18.390	22.700	25.590
TRM58994	20.990	13.610	16.440	20.690	18.140	15.490	20.950	12.080	18.080	14.520	18.490	14.730
TRM58993	13.370	-	-	-	11.680	10.940	12.080	-	11.780	-	-	11.340
TRM58992	22.250	16.960	15.320	18.240	19.320	19.100	17.050	15.900	23.830	16.710	19.820	16.720

Note:EC, *Escherichia coli* ACTC25922; Sal, *Salmonella enteritidis* ACTCC 25923; Sal D58-1, Sal D58-2, Sal D62-3, and Sal F21 indicate *Salmonella enteritidis* D58-1, D58-2, D62-3, and F21, respectively; Pa, *Pseudomonas aeruginosa* ACTCC 27853; Kp, *Klebsiella pneumoniae* ATCC 10031; Ab, *Acinetobacter baumannii* ACTCC 19606; Sf, *Shigella flexneri* ATCC 12022; Sa, *Staphylococcus aureus* ACTCC 6538; and Ca, *Candida albicans* ATCC 10231; -: No antibacterial activity.

*Antibiotic sensitivity and safety* (Table 5) reveals that 8 strains are resistant to SXT, while 6 strains demonstrate sensitivity to at least four antibiotics and exhibit multi-drug resistance to two or more antibiotics. Significantly, none of the 8 strains exhibited a hemolytic reaction, indicating the absence of virulence and affirming the biological safety of these lactic acid bacteria.

**Table 5:** Sensitivity of lactic acid bacteria to antibiotics and hemolytic assay.

Susceptibility	Antibiotics										Hemolytic
	CI	CTR	AMP	E	C	PEN	TET	GEN	MY	SXT	
TRM58999	I	R	R	R	S	S	S	R	R	R	-
TRM58998	I	R	S	I	S	S	S	R	R	R	-
TRM58997	I	I	S	R	I	R	R	R	R	R	-
TRM58996	S	S	S	R	I	S	R	R	R	R	-
TRM58995	R	I	S	S	S	S	S	R	S	R	-
TRM58994	R	R	R	R	R	R	R	R	R	R	-
TRM58993	R	I	S	I	S	S	S	I	R	R	-
TRM58992	R	I	R	R	R	R	R	R	R	R	-

Note:CI, Ciprofloxacin; CTR, Ceftriaxone; AMP, Ampicillin; E, Erythromycin; C, Chloramphenicol; PEN, Penicillin; TET, Tetracycline; GEN, Gentamicin; MY, Lincomycin; SXT, Compound Sulfamethoxazole.

R: Drug resistance; I, Neutral sensitivity; S, Sensitivity; -, No hemolytic ring.

### Animal feeding experiment

*Production Performance and Egg Quality* Quails supplemented with TRM58998 demonstrated a significantly higher egg production compared to the control group (P < 0.05). Additionally, they exhibited a significantly improved feed-to-egg ratio (P < 0.05), although their average daily feed intake from weeks 1 to 4 remained unaffected (P > 0.05) (Table 6).

**Table 6:** The influence of probiotics on the performance of laying quail.

Items	Treatments		p-Value
	Control	TRM58998	
Egg production, %			
Week1	75.595 ± 0.019	81.548 ± 0.015	0.032
Week2	75.595 ± 0.023	81.548 ± 0.012	0.048
Week3	75.298 ± 0.013	82.143 ± 0.015	0.005
Week4	70.833 ± 0.018	81.548 ± 0.012	<0.001
Average egg weight, g/ egg			
Week1	10.231 ± 0.090	10.664 ± 0.045	0.005
Week2	9.991 ± 0.050	11.164 ± 0.141	<0.001
Week3	10.032 ± 0.072	10.949 ± 0.069	<0.001
Week4	10.137 ± 0.071	10.882 ± 0.209	0.015
Average daily feed intake, g/per day			
Week1	24.935 ± 0.030	24.982 ± 0.008	0.170
Week2	24.893 ± 0.037	24.964 ± 0.011	0.109
Week3	24.940 ± 0.034	24.982 ± 0.012	0.270
Week4	24.940 ± 0.027	24.982 ± 0.008	0.167
Feed-to-egg ratio			
Week1-4	2.478 ± 0.023	2.276 ± 0.024	<0.001

Regarding egg quality, there were no significant differences in the egg shape index, yolk index, or yolk ratio at the end of the experiment (P > 0.05). However, eggshell strength and thickness increased significantly (P < 0.001), while the Haugh unit showed improvement without significant trend changes (P > 0.05) (Table 7).

**Table 7:** The impact of probiotics on the egg characteristics of laying quails.

Items	Treatments		p-Value
	Control	TRM58998	
Egg-shaped index	1.254 ± 0.012	1.244 ± 0.012	0.563
Egg yolk index	0.450 ± 0.010	0.469 ± 0.008	0.158
Yolk proportion	0.311 ± 0.004	0.307 ± 0.004	0.495
Eggshell strength(N)	1.535 ± 0.003	1.578 ± 0.005	<0.001
Eggshell thickness(mm)	0.192 ± 0.000	0.201 ± 0.000	<0.001
Haugh unit	85.948 ± 0.529	86.605 ± 0.446	0.346

*Serum biochemical indexes* Compared with the control group, the concentration of GLOB and TP in TRM58998 group was increased (P = 0.028, P = 0.004). The concentrations of TB and A/G were not altered (P > 0.05), and the GLU concentration was reduced compared to the control group, but it was not statistically significant (P > 0.05). The concentration of other indicators was slightly higher than of the control group, but not obvious (P > 0.05) (Table 8).

**Table 8:** Impact of probiotics on serum biochemical indices during the late egg-laying period.

Items	Control	TRM58998	p-Value
Ca	3.540 ± 0.215	4.387 ± 0.269	0.070
PHOS	3.507 ± 0.306	5.030 ± 0.843	0.165
Ca:PHOS	12.330 ± 0.752	15.640 ± 2.400	0.259
AMY	234.667 ± 24.700	480.33 ± 130.825	0.139
TB	3.700 ± 0.252	3.730 ± 0.338	0.941
TG	6.120 ± 0.780	7.657 ± 1.357	0.382
GLU	11.020 ± 1.222	6.790 ± 2.910	0.251
BUN	1.280 ± 0.181	1.473 ± 0.095	0.398
CK	2072.000 ± 162.235	2244.330 ± 87.592	0.403
Crea	<10.000	<10.000	-
ALT	20.000 ± 13.528	28.330 ± 10.414	0.651
TP	41.000 ± 0.643	52.130 ± 1.748	0.004
ALB	15.000 ± 0.361	17.500 ± 1.442	0.168
GLOB	26.000 ± 0.351	34.830 ± 2.583	0.028
A/G	0.577 ± 0.009	0.577 ± 0.032	1.000

Note: ALB, Albumin; TP, Total protein; GLOB, Globulin; ALT, Alanine aminotransferase; AST, Aspartate aminotransferase; BUN, Urea nitrogen; AMY, amylase; ALP, Alkaline phosphatase; Crea, Creatinine; GLU, Glucose; TG, Triglycerides; TC, Total cholesterol; Ca, Calcium; PHOS, Inorganic phosphorus.

*Serum Antioxidant Capacity* The addition of TRM58998 could increase the trend of serum CAT concentration (P = 0.005). It could increase the concentration of serum SOD and there was a tendency to reduce serum MDA concentration, but the effect was not obvious (P > 0.05), Finally, The concentrations of GSH-Px were not altered (P > 0.05) (Table 9).

**Table 9:** Impact of TRM58988 on the blood antioxidant capacity of quail during the late stage of egg production.

Items	Control	TRM58998	p-Value
MDA (nmol/mL)	0.481 ± 0.029	0.434 ± 0.064	0.510
SOD (U/mL)	14.881 ± 0.641	16.962 ± 0.850	0.079
GSH-Px (nmol/min.mL)	183.743 ± 4.510	187.018 ± 5.031	0.634
CAT (μmol/min/mL)	15.754 ± 1.019	21.119 ± 1.273	0.005

*Hormonal Levels and Follicle Development* The supplementation of *E. lactis* significantly enhanced reproductive hormone levels, with higher concentrations of FSH (P = 0.017), LH and E2 detected in the treatment group (Table 10). These hormonal changes are crucial for regulating follicular development and ovulation, ensuring consistent egg production. Additionally, the number of pre-ovulatory follicles (≥12 mm) was significantly greater in the treatment group (Table 11), indicating improved ovarian function.

**Table 10:** Influence of TRM58988 on serum hormone levels in quail during the critical late stages of egg production.

Items	Control	TRM58998	p-Value
FSH (U/L)	9.880 ± 0.210	10.555 ± 0.141	0.017
LH (pg/mL)	69.388 ± 2.045	68.773 ± 2.559	0.853
E2 (pmol/L)	98.627 ± 4.423	106.373 ± 4.246	0.223

**Table 11:** Impact of TRM58988 on follicle count in quail during late egg production stage.

Items	Control	TRM58998	p-Value
Preovulatory follicles	1.625 ± 0.183	2.000 ± 0.000	0.060
Small yellow follicles	1.500 ± 0.189	1.750 ± 0.164	0.334
Large white follicles	14.500 ± 0.378	18.250 ± 0.559	<0.001

**Fig. 2:**
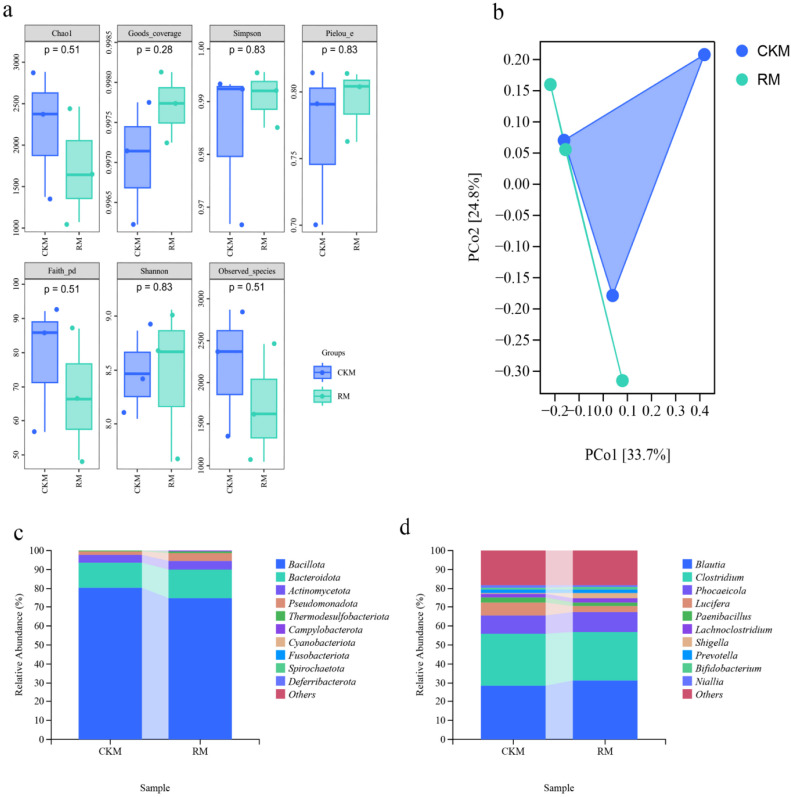
a, RM represents TRM58998, while CKM represents the normal feeding group. a diversity index was used for analysis. b, The PcoA composition was also analyzed. c, The difference in relative abundance between the two groups of cecal microbial communities was examined at the phylum level. d, Differences in the relative abundance of cecal microbial communities between the two groups were also analyzed at the genus level.

*Analysis of the genus-level structural composition of the cecal colony* The 16S rDNA sequencing analysis revealed that the probiotic supplementation resulted in significant shifts in the cecal microbiota composition. In the treatment group, the relative abundance of beneficial bacteria, such as *Blautia* and *Clostridium*, increased (P > 0.05) (Fig. 2). Although alpha and beta diversity indices did not show significant differences between the two groups, the increase in beneficial bacteria suggests that *E. lactis* promoted a healthier microbial environment. A well-balanced microbiota plays a key role in enhancing nutrient absorption and strengthening immune responses, which further supports the improved performance observed in the treatment group.

**Fig. 3:**
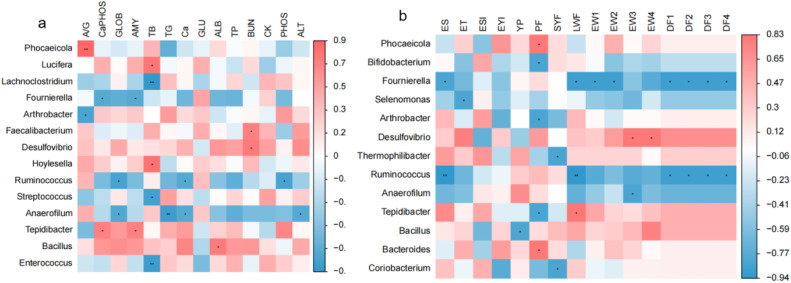
a, represents the correlation between intestinal microbiota and serum biochemical indicators, b, represents the correlation between egg quality and laying performance, where ES: Eggshell strength, ET: Eggshell thickness, ESI: Egg-shaped index, EYI: Egg yolk index, YP: Yolk proportion, PF: Preovulatory follicles, SYF: Small yellow follicles, LWF: Large white follicles, EW1-4: Egg production, DF1-4: Average daily feed intake, g/per day.

Correlations between gut microbiota abundance and factors such as blood biochemical indices, egg quality, and laying performance of quail were analyzed using Spearman correlation analysis. As shown in Fig. 3: (a) For serum biochemistry, TB levels exhibit a negative correlation with *Lachnoclostridium, Streptococcus*, and *Enterococcus*, while showing a positive correlation with *Lucifera* and *Hoylesella*. GLOB levels are negatively correlated with *Ruminococcus* and *Anaerofilum*. (b) Regarding egg quality and laying performance, ES levels are negatively correlated with *Fournierella* and *Ruminococcus*. PF levels positively correlate with *Phocaeicola* and *Bacteroides*, but negatively correlate with *Bifidobacterium* and *Arthrobacter*. Additionally, *Fournierella* and *Ruminococcus* negatively affect average egg weight and daily feed intake.

## Discussion

This study investigated the impact of dietary supplementation with *E. lactis* TRM58998 on the performance, egg quality, antioxidant capacity, reproductive function, and intestinal health of quails during the late egg-laying period. The findings demonstrate multiple beneficial effects of *E. lactis*, supporting its role as an effective dietary supplement for quails, in line with previous research on probiotics in poultry nutrition (Dong et al., 2022; Rehman et al., 2020).

The improved growth performance observed in the treatment group, as reflected in increased egg production and reduced feed conversion ratio (FCR), suggests that the probiotic enhanced nutrient absorption and utilization. This aligns with previous studies showing that probiotics improve feed efficiency by enhancing intestinal function and nutrient uptake (Rehman et al., 2020). Furthermore, the ability of the treatment group to maintain productivity during the late laying period highlights the role of probiotics in mitigating stress-related declines in performance, which is consistent with findings by Shanmugam et al (Shanmugam et al., 2024), who reported that probiotics increased egg weight and reduced breakage in older hens.

Egg quality, especially shell strength and thickness, was significantly enhanced in the treatment group. This suggests that *E. lactis* may have facilitated calcium metabolism, which is crucial for maintaining eggshell integrity, particularly during the late laying period when calcium depletion is common (Fu et al., 2024). Similar benefits were reported by Nour et al (Nour et al., 2021), who observed improvements in egg quality following probiotic supplementation in quails. Although the increase in Haugh units was not statistically significant, the trend suggests that probiotic supplementation helped maintain albumen quality, which is essential for preserving egg freshness (Lokapirnasari et al., 2019).

The serum biochemical analysis revealed that *E. lactis* supplementation increased TP, ALB, and GLOB levels, indicating improved metabolic and immune function. These results align with prior studies that demonstrated the ability of probiotics to enhance protein metabolism and immune responses in poultry. Additionally, elevated levels of antioxidant enzymes, such as CAT and SOD, along with reduced MDA levels, suggest that *E. lactis* alleviated oxidative stress, consistent with findings by Hui et al (Hui et al., 2024) in pigs. The ability to mitigate oxidative damage is critical during the late laying period, when birds are more susceptible to stress-related declines in productivity.

The enhanced reproductive performance observed in the treatment group, reflected in higher levels of FSH, luteinizing hormone LH, and E2, as well as an increased number of pre-ovulatory follicles, indicates that *E. lactis* positively influenced ovarian function. These findings are consistent with those of Shi et al (Shi et al., 2024), who demonstrated that probiotics help maintain high egg production by supporting reproductive health in laying hens. The improved reproductive function in this study may be attributed to enhanced nutrient absorption and reduced oxidative stress, which collectively support follicular development and ovulation (Wu et al., 2022; Xu et al., 2023).

The analysis of cecal microbiota revealed significant changes in microbial composition following *E. lactis* supplementation. The increased abundance of beneficial bacteria, such as *Blautia* and *Clostridium*, and the reduction of harmful pathogens, including *Escherichia coli* and *Salmonella enteritidis*, are consistent with previous studies demonstrating that probiotics promote beneficial bacteria while inhibiting pathogenic species (Dong et al., 2022; Xu et al., 2023). Although alpha and beta diversity indices did not show significant differences, the shift toward a healthier microbial composition likely contributed to improved nutrient absorption and immune function, further supporting overall bird performance during the late egg-laying period (Shu et al., 2024).

These findings highlight the potential of *E. lactis* TRM58998 as a valuable dietary supplement for improving quail productivity and health. The results align with existing literature on the benefits of host-derived probiotics, which offer enhanced adaptability and effectiveness compared to non-host-specific strains (Rehman et al., 2020). Incorporating *E. lactis* into quail diets provides a sustainable strategy to support growth, reproductive function, and egg quality, especially during challenging physiological stages such as the late egg-laying period.

Future studies should explore the long-term effects of *E. lactis* supplementation and investigate potential synergies with other natural additives, such as prebiotics or organic acids. Further research into the molecular mechanisms underlying the observed benefits will provide deeper insights into how *E. lactis* interacts with host physiology, supporting the development of optimized probiotic formulations tailored to the needs of poultry.

## Conclusions

The results of the study showed that a total of eight LABs were isolated from quail cecum and feces, among which the isolate TRM58998, identified as *E. lactis*, outperformed the other strains in terms of in vitro probiotic properties (acid and bile salt resistance, bacteriostatic ability). TRM58998 was fed to 43-week-old quail at a dose of 1 × 10^8^ CFU/g for 30 days, and the results showed that TRM58998 had a positive effect on laying performance, total egg production, antioxidant capacity, reproductive health, and intestinal microbiota in the late-stage of quail laying.

## Author contributions

Lan Liu, Changcai Liu, and colleagues were responsible for the research design and implementation. Lan Liu and Changcai Liu conducted the data analysis and contributed to manuscript preparation, while Min Ren supervised the project.

## Declaration of competing interest

The authors declare the following financial interests/personal relationships which may be considered as potential competing interests:

Min Ren reports equipment, drugs, or supplies was provided by Xinjiang Aksu region Wensu County moss flower farming and animal husbandry technology Co., Ltd. If there are other authors, they declare that they have no known competing financial interests or personal relationships that could have appeared to influence the work reported in this paper.
